# Gas Diffusion Layer for Proton Exchange Membrane Fuel Cells: A Review

**DOI:** 10.3390/ma15248800

**Published:** 2022-12-09

**Authors:** Hui Guo, Lubing Chen, Sara Adeeba Ismail, Lulu Jiang, Shihang Guo, Jie Gu, Xiaorong Zhang, Yifeng Li, Yuwen Zhu, Zihan Zhang, Donglin Han

**Affiliations:** 1 College of Energy, Soochow University, No 1 Shizi Street, Suzhou 215006, China; 2 Provincial Key Laboratory for Advanced Carbon Materials and Wearable Energy Technologies, Soochow University, No 1 Shizi Street, Suzhou 215006, China; 3 Light Industry Institute of Electrochemical Power Sources, Shahu Science & Technology Innovation Park, Suzhou 215638, China; 4 Key Laboratory of Core Technology of High Specific Energy Battery and Key Materials for Petroleum and Chemical Industry, Soochow University, Suzhou 215006, China

**Keywords:** proton exchange membrane fuel cell, gas diffusion layer, macroporous substrate, microporous layer, water management, gas diffusion, carbon fiber

## Abstract

Proton exchange membrane fuel cells (PEMFCs) are an attractive type of fuel cell that have received successful commercialization, benefitted from its unique advantages (including an all solid-state structure, a low operating temperature and low environmental impact). In general, the structure of PEMFCs can be regarded as a sequential stacking of functional layers, among which the gas diffusion layer (GDL) plays an important role in connecting bipolar plates and catalyst layers both physically and electrically, offering a route for gas diffusion and drainage and providing mechanical support to the membrane electrode assemblies. The GDL commonly contains two layers; one is a thick and rigid macroporous substrate (MPS) and the other is a thin microporous layer (MPL), both with special functions. This work provides a brief review on the GDL to explain its structure and functions, summarize recent progress and outline future perspectives.

## 1. Introduction

Energy development is a deep focus in any era. Traditional fossil fuel energy is now inefficient on a worldwide scale, which has resulted in various degrees of pollution and harm to the environment. In this context, the development of efficient and clean energy resources has generally become the consensus of all countries. Since William Robert Grove, the father of fuel cells, reported the development of the first fuel cell in the world, fuel cells have gone through hundreds of years of history [1]. Fuel cells use fuels and oxygen (or air) to release electrical energy directly via electrochemical reactions [2,3]. Unlike secondary batteries, which require recharging, fuel cells can produce electricity continuously as long as there is a supply of fuels and oxygen. This is superior to the intermittent behavior of other renewable energy sources, such as solar power and wind power [4]. Additionally, the efficiency of fuel cells is much higher than that of other power generation systems because there is no heat exchange and mechanical transmission process. The fuels used in the fuel cells mainly include hydrogen, methane, methanol, ethanol and other clean energy [5,6,7,8,9]. There is no combustion in the whole working process, so its energy conversion efficiency is not limited by the Carnot cycle, giving rise to a high specific energy.

Depending on the difference in electrolytes, fuel cells can be mainly divided into alkaline fuel cells (AFCs), molten carbonate fuel cells (MCFCs), proton exchange membrane fuel cells (PEMFCs), solid oxide fuel cells (SOFCs) and phosphoric acid fuel cells (PAFCs) [10]. Among them, PEMFCs are well developed in technique and are commercially applicable. PEMFCs mainly use perfluorinated polymers (e.g., Nafion) as electrolytes, and as a result they are not tolerant to high temperature and typically work at around 80 °C. Catalysts containing novel metals (e.g., Pt/C) are therefore required to obtain sufficient activity for the oxygen reduction reaction (ORR) and the hydrogen oxidation reaction (HOR) at the cathode and anode, respectively. Due to the advantages of a simple structure, fast start-up at room temperature, a long life and high energy density, PEMFCs are particularly suitable for applications in automobiles, distributed power plants and portable electronic devices [11,12,13]. Currently, fuel cell vehicles (FCVs)—mainly using PEMFCs—are essential for providing low-carbon transport. Moreover, by using the hydrogen produced from renewable energy as the fuel, FCVs emit almost zero greenhouse gas. The key component of PEMFCs is the membrane electrode assembly (MEA), which is a sequential stack of an anode gas diffusion layer (GDL), an anode catalyst layer (CL), an electrolyte layer, a cathode CL and a cathode GDL. The GDL provides the route for gas diffusion and drainage and electrical connection between the CL and bipolar plate (BP) while also supporting the MEA mechanically. Macroporous substrate (MPS) takes up the main volume of MPS, but to further improve the performance of the GDL, the interface between the GDL and the CL has to be handled carefully, leading to the insertion of a microporous layer (MPL). Recently, significant progress has been achieved on the development of the GDL and, in particular, the MPL. Therefore, in this work, we conducted a review on GDL and placed special attention on the progress of the processes of fabrication and hydrophobization for the MPL.

## 2. Brief Introduction of PEMFCs

**Figure 1:**
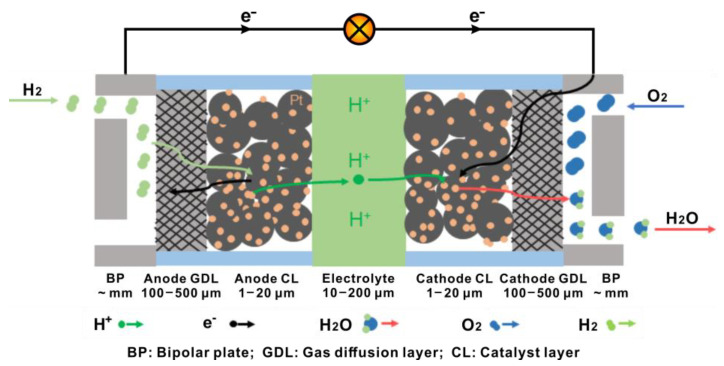
Schematic diagram of the structure of a PEMFC.

A general structure of PEMFCs is shown in Figure 1. The main role of BPs is to conduct electrons and transport reactants and products through the gas channels [14]. Currently, the most common materials used for BP are graphite, corrosion-resistant metals and carbon-based or metal-based composites [15]. The flow field design of the BPs determines the diffusion and distribution of the gas in PEMFCs, as well as the rate of chemical reactions, the distribution of current density and the removal of liquid water [16]. The GDL mainly plays a role supporting the cell structure and transmitting electrons, reactants, products and heat [17]. At present, carbon fiber paper and cloth are the GDL materials with developed applications and an extensive research base. In order to obtain sufficient electrode reactivity at the low operating temperature of PEMFCs, catalysts containing Pt (e.g., Pt/C) are commonly used in the CL. Moreover, some alloy catalysts like PtRu/C and SnPt/C also show excellent carbon monoxide tolerance and are suitable for application in direct methanol or ethanol PEMFCs [18,19]. The electrolytes used are commonly perfluorinated polymers, which are proton-conducting and electron-insulating and act as a separator for the reactants and the products between the cathode and anode as well [20].

Referring to the working mechanism of PEMFCs, as shown in Figure 1, hydrogen reaches the anode CL by passing through the anode GDL and dissociates into protons and electrons, following

(1) $$H_{2}\;\to {\;2H}^{+}{+2e}^{-}$$

Protons move across the electrolyte and react with oxygen gas at the cathode CL, following

(2) $$O_{2}{+4H}^{+}{+4e}^{-}\;\to {\;2H}_{2}O+\mathrm{Heat}$$

The GDL at the cathode side leads the oxygen passing through it to the cathode CL and expels the water produced from the cathode CL out of the system. Since the operating temperature of PEMFCs is usually low (typically around 80 °C), water generated at the cathode is easy to condense into liquid water. If this liquid water is not drained away in time, it occupies the open space in the cathode CL and GDL and causes flooding. This blocks the transport channels for oxygen, leading to a limited current and a dramatic degradation in the performance of PEMFCs during operation [21,22,23].

## 3. General Requirements for GDL

The GDL is one of the core components of PEMFCs, located between and connecting the BP and CL. It provides support not only for the MEA, but also for the route for reactants and drainage, and it has the following requirements.

(1) High gas permeability. The pore structure of GDL impacts the transport properties greatly, since the gas involved in the reaction needs to pass through the GDL to reach the CL to participate in the electrochemical reaction. The electrolyte membrane also needs to be hydrated by being subjected to the wet atmosphere to maintain high proton conductivity. Moreover, water generated from the reaction in PEMFCs needs adequate routes to be drained without flooding the CL and GDL [24,25,26]. Zhou et al. [27] found that the ability of mass transfer was enhanced by reducing the length of the channel for gas flow and increasing the porosity of the anode GDL (A-GDL). Sim et al. [28] reported a new approach to increasing the porosity and pore size of GDL by changing the gasket thickness, resulting in a positive effect on the drainage capacity.
(2) High electrical conductivity. The GDL provides the electrical connection between the CL and the BP. It delivers electrons from the BP to cathode CL, and collects electrons from anode CL to the BP. Therefore, high electrical conductivity is required for a GDL to decrease its ohmic loss and contact resistance with the adjacent BP and CL [29].
(3) High thermal conductivity. During the operation of PEMFCs, heat is generated and tends to accumulate locally. If the extra heat is not removed quickly from the system, the increasing temperature shortens the longevity of PEMFCs. Thereby, the GDL must have high thermal conductivity to conduct the extra heat quickly to the BP to ensure a safe system temperature. Botelho et al. [30] applied a resistance network theory to estimate effective thermal contact resistance between the carbon fibers used for composing the GDL. They found that the change in fiber roughness led to a large change in the effective thermal contact resistance, implying a feasible approach to regulate the thermal conduction of the GDL by controlling the morphology of the carbon fibers.
(4) Good mechanical strength. The GDL must be robust enough to offer mechanical support to the MEA and protect the CL and the electrolyte membrane. Furthermore, a mechanically stable porous structure is vital to construct and maintain channels for gas diffusion and drainage. Csoklich et al. [31] suggested that it was efficient to improve the performance of the GDL by optimizing the structure rather than improving the thermal conduction in order to achieve excellent performance at high current density.
(5) Good chemical/thermal stability and corrosion resistance. To minimize the degradation rate during long-term operation, the GDL is required to be stable both chemically and thermally, as well as corrosion-resistant in both oxidizing and reducing atmospheres in the chambers of cathode and anode, respectively.
(6) Facilitation of water removal. To facilitate the drainage at the cathode, the GDL at the cathode is usually processed to be hydrophobic. Moreover, other factors (such as the geometric parameters of the carbon fibers composing the GDL) also effect the hydrophobicity. Wang et al. [32] found that the speed of water discharge was enhanced by increasing the diameter of the carbon fibers in the GDL.
(7) Low cost. The volumetric ratio of the GDL in PEMFCs is not low (larger than that of the CL and electrolyte), so it is necessary to choose proper materials with both a low cost and a high performance.

## 4. Macroporous Substrate

A GDL has generally a bilayer structure composed of a thick macroporous substrate (MPS)—the basis of the GDL—and a thin microporous layer (MPL). Carbon fiber-based (e.g., carbon fiber paper and cloth) and metal-based (e.g., metal mesh and porous metal foam) materials are candidates for the MPS. Although the metal-based materials have good mechanical properties, they face substantial challenges with corrosion and are also too hydrophilic, so carbon-based materials are currently the mainstream choice for the MPS [33]. The carbon-based materials used have high electrical and thermal conductivity, excellent resistance against electrochemical corrosion and low cost. The channels for gas flow and drainage in the MPS can be constructed by stacking carbon fibers to form porous carbon fiber paper and cloth.

### 4.1. Carbon Fiber Paper

**Figure 2:**
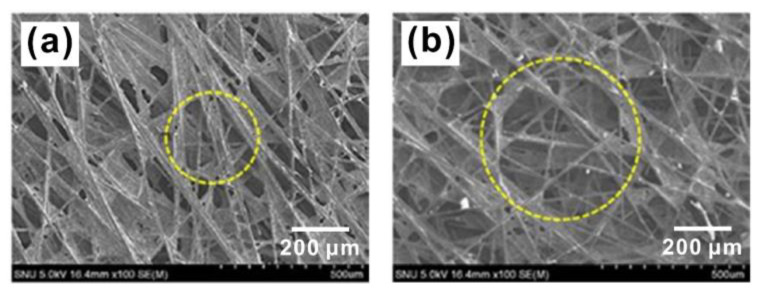
SEM images of carbon paper made from (**a**) short fibers and (**b**) long fibers. Yellow circles highlight the pore formed among the fibers. Reproduced with permission [41]. Copyright 2016, Elsevier.

Carbon fiber paper is the most widely used material for MPS due to its ease in shaping a thin layer with a uniform porous structure, its high electrical/thermal conductivity and its excellent chemical/thermal stability [34,35,36]. The carbon fibers used in MPS are generally 5–15 μm in diameter, and the thickness of the carbon fiber paper thereby prepared typically has a thickness within 200–400 μm and an average pore size of tens of micrometers [37,38,39]. Interestingly, the morphology and microstructure and, thereby, the mechanical/electrical properties of the carbon fiber paper appear to be influenced significantly by the mass ratio and the geometric parameters of the carbon fibers. Kitago et al. [40] studied the effect of the mass ratio of carbon fibers in pulp on the morphology of the as-prepared carbon fiber paper and found that when the content of carbon paper was too low, wrinkles formed on the surface of the carbon fiber paper during the carbonization process, leading to decreased MPS strength. However, if the content of carbon fiber was too high, the density of the carbon fiber paper decreased. Park et al. [41] used carbon fibers with different lengths to compose a carbon fiber paper and compared their effects on the performance of PEMFCs. They found that longer carbon fibers led to a large pore size, as shown in Figure 2, benefiting drainage. However, it also resulted in rough surficial morphology, giving rise to high contact resistance. Such results indicate the importance of controlling the pore size or the distribution of the pores with different sizes to satisfy specific requirements of water management and electricity collection and grant the possibility to control the electrochemical and physicochemical properties of MPS by choosing proper raw materials to construct the carbon fiber paper.

### 4.2. Carbon Fiber Cloth

**Figure 3:**
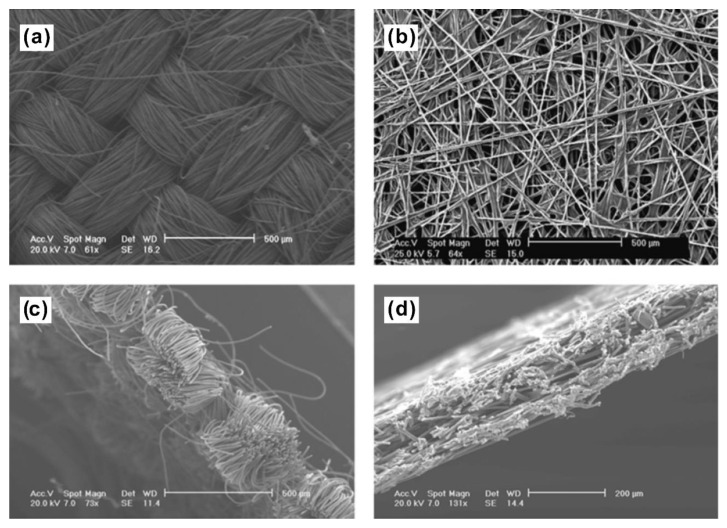
SEM images of the configurations of fibers in (**a**,**c**) carbon fiber cloth (Ballard 1071HCB), and (**b**,**d**) carbon fiber paper (Toray H-060) [46].

Carbon fiber cloth is also widely adopted as the preeminent MPS material due to its high gas permeability and electrical/thermal conductivity [42]. Compared with the carbon fiber paper, there are fewer fixed junctions among the fibers in the carbon fiber cloth (a comparison between the carbon fiber cloth and paper is given in Figure 3), making it thereby easier to deform and stretch and thus fit better with the surface of the CL and BP [43]. However, the electrical conductivity of the carbon fiber cloth is lower than that of the carbon fiber paper. With the aim to increase the conductivity and improve the rigidity of carbon fiber cloth, some additives (like carbon black powder) are usually added into the carbon fiber cloth [42]. Along with this, a similar effect can also be achieved by coating the carbon fibers with phenolic resin, followed with a subsequent high temperature carbonization process [44]. Yang et al. [45] prepared a polyacrylonitrile (PAN)-derived carbon cloth showing low sheet resistance and high chemical stability. They controlled the fabric count and yarn plies to produce the carbon cloth precursor by oxidizing the PAN-based fiber, which was finally graphitized via annealing in Ar at 1700 °C. The as-prepared carbon fiber cloth contained 98.24% carbon (the other elements were H, S and O) and had an in-sheet resistance of 820 mΩ/□. The Raman spectra showed that the carbon cloth graphitized at 1700 °C had higher integrated intensities of peaks in 1360 cm^−1^ and 1580 cm^−1^ than that graphitized at lower temperature, indicating an enhanced graphite structure ratio with an increasing temperature of graphitization.

## 5. Microporous Layer

**Figure 4:**
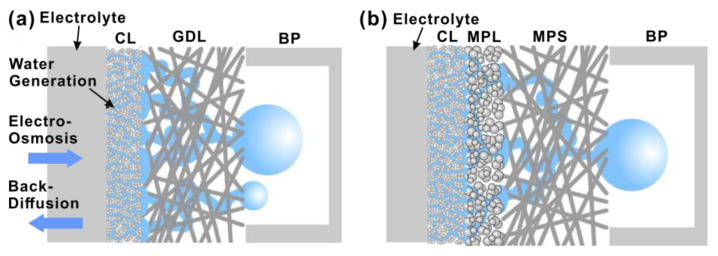
Schematic illustrations showing water transport in the cathode: (**a**) without and (**b**) with the MPL. Reproduced with Permission [51]. Copyright 2009, Elsevier.

To improve the interface between the GDL with large-scale porosity (pore size of 10–30 μm) and the CL with fine-scale porosity (pore size of 10–100 nm), an intermediate microporous layer (MPL) with a pore size around 0.1–20 nm is commonly inserted, reducing the contact resistance between the GDL and CL and providing good mechanical support and protection for the CL [47,48,49]. Aside from this, the MPL also improves the wicking of liquid water from CL and, therefore, the drainage of the cathode, as shown in Figure 4. The properties of MPL are mainly affected by the size and distribution of pores, hydrophobicity and conductivity, which are closely related to the materials and preparation methods used for the MPL [50].

The MPL is mainly composed of conductive carbon materials and a hydrophobic agent (usually PTFE). Common carbon materials include carbon black, graphite and carbon nanotubes (CNTs), etc. [52,53,54,55,56]. The conductive carbon materials enhance the conductivity of the MPL, while the hydrophobic agent improves the hydrophobicity [57]. The pore size of the MPL is much smaller than that of the MPS, so the airflow entering the CL through the MPL can be rectified with an improved uniformity. The capillarity effect of the micropores in the MPL further helps in removing water generated from the cathode reaction [58].

The selection of conductive carbon materials and the hydrophobic agent has a great effect on the performance of an MPL. Li et al. [54] performed a quantitative analysis on the properties of MPLs prepared from different carbon materials and the same hydrophobic agent and found that the hydrophobicity differed between MPLs prepared with different carbon materials. The distribution of micropore in the MPLs prepared from the different carbon materials was different, and the micropores in the MPL usually transported water through a capillarity effect, resulting in the difference in the hydrophobicity of MPLs. Such results imply that the hydrophobic agent is not the only factor influencing the hydrophobicity of MPL. The carbon materials chosen for preparing the MPL also play a vital role. Xie et al. [59] incorporated CNTs into the MPL via a plasma enhanced chemical vapor deposition (PECVD) process and succeeded in preparing a MPL with an appropriate hydrophobicity and pore structure. Graphene is an attractive member of the carbon material family and was also added into the MPL due to its excellent electrical and thermal conduction. Ozden et al. [60] found that by adding graphene into an MPL, the contact resistance between the MPS and the CL was reduced, and the in-plane conductivity was also increased. Moreover, such a graphene-added MPL showed positive effect on preventing the catalyst particles entering GDL, enabling better utilization of the catalysts.

### 5.1. MPL Fabrication Process

A conventional approach to preparing an MPL is mixing the conductive carbon materials, hydrophobic agents (such as PTFE, etc.), solvents (such as deionized water, isopropanol, etc.) and/or surfactants to obtain a well dispersed MPL slurry, which is then deposited onto the MPS by spraying, scraper coating or screen printing with subsequent drying and/or sintering steps [61,62,63,64]. Generally, carbon black, PTFE and ethanol solvent are mixed to form a solution, which is then sprayed onto the MPS via spraying. Li et al. [61] stacked two MPLs with the same thickness and different pore size by spraying the solutions with different content of CaCO_3_ as the pore-forming agent. The as-prepared MPL exhibited a gradient distribution of pore size along the thickness direction, improving the water/vapor management capabilities of the fuel cells. The scraper coating method mainly uses high viscosity ink, which is coated onto the substrate with a blade. In this process, the ink viscosity, coating speed and the distance between the blade and the substrate affect the coating thickness [63]. The screen-printing method is used to prepare the MPL via scraping and printing slurries containing carbon materials and an aqueous agent on the substrate with a silk screen and a scraper, enabling uniform deposition of the MPL with large area and controllable patterns. Yan et al. [64] compared the differences between the MPLs prepared by screen-printing and spray coating, and found that the fractures in the screen-printed MPL are smaller than those of the spray-deposited MPL. A possible reason for this may be that the screen-printing method controls the thickness and uniformity of the as-prepared MPL better than the spraying method. Due to the existence of fractures, the permeability of the spray-deposited MPL was higher than that of the screen-printed one. Moreover, the contact resistance between the screen-printed MPL and the CL was lower, due to the larger contact area. Therefore, the authors concluded that the screen-printed MPL had better performance.

Besides the conventional approaches to preparing MPL, chemical vapor deposition (CVD) is also adopted for preparing MPL. In the work of Xie et al. [59], carbon paper is infiltrated with ethanol solution mixed with Ni(NO_3_)_2_ with a subsequent drying process at 80 °C. Then, the carbon paper is annealed in hydrogen plasma at 800 °C under 40 W microwave power for 5 min. Finally, the CNTs were synthesized in mixed precursors (CH_4_/H_2_: 80/20) at a microwave power of 200 W under a chamber pressure of 1.2 kPa for 30 min. Kannan et al. [65] compared the pore size distribution of an in-situ grown CNT-based MPL and wire-rod coated MPL, and observed that the in-situ grown CNT-based MPL only showed large pores with a diameter of 30 μm, leading to better gas diffusion. The wire-rod coated MPL had pores with different scales (diameters of 0.075, 5, 40 and 225 μm). The fuel cells using such a CNT-based MPL therefore showed more stable performance under different humidity conditions. Moreover, the CNTs grown through the CVD method exhibited high electrical conductivity and inherent hydrophobicity, reducing the use of PTFE in the preparation process [66].

In addition, electrospinning is also applied to prepare the MPL. PAN (or its mixture with polyvinyl pyrrolidone (PVP) and polyimide (PI)) were used as raw materials to prepare polymer nanofiber felt via the electrospinning method. Then, they were stabilized and carbonized to form a carbon nanofiber (CNF) felt, which is also known as fiber layout type MPL. By controlling the temperature in the carbonization process, the non-conductive polymer nanofibers can be transferred to a conductive CNF with a controllable degree of graphitization [67]. The fibers in the electrospun MPL do not penetrate into MPS, giving rise to the reducing gas barrier and good water management [68].

### 5.2. Hydrophobization

Besides PTFE, which is the main hydrophobic agent for MPL, there are some other candidates, including polydimethylsiloxane (PDMS), fluorinated ethylene (FEP), perfluoropolyether (PFPE) and poly(vinylidene) fluoride (PVDF) [57,69,70,71,72]. The hydrophobic agent is commonly non-conductive, so the content of the hydrophobic agent must be controlled carefully to make the MPL have both high electrical conductivity and hydrophobicity.

**Figure 5:**
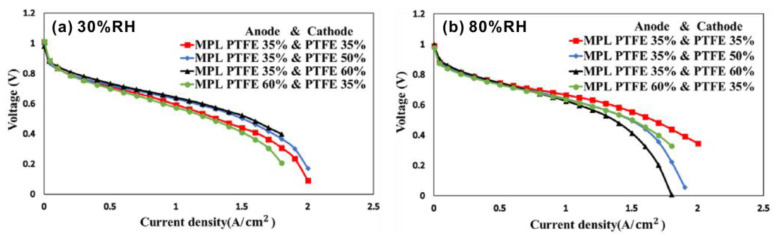
I–V curves of the PEMFCs with different PTFE content in the GDLs in anodes and cathodes at specific relative humidity of (**a**) 30%, and (**b**) 80%. Reproduced with permission [73]. Copyright 2021, Elsevier.

As shown in Figure 5, the content of PTFE in both the GDLs in anodes and cathodes influences the performance of PEMFCs significantly [73]. At a low relative humidity (RH) of 30%, the performance of PEMFCs was improved when the PTFE content in the cathode GDL increased from 35% to 60%, with the PTFE content in the anode GDL kept at 35%, due to the enhanced repulsive force between the GDL and liquid water due to the increasing PTFE content. However, at a higher RH of 60%, the increasing PTFE content in the cathode GDL resulted in degradation of the performance of PEMFCs. This may be due to the decreasing porosity and increasing tortuosity of the GDL with the increasing PTFE, giving rise to the decreasing drainage capacity of the GDL at a high relative humidity [73]. Giorgi et al. [74] studied the case of high current density of 2.3 ± 0.2 Acm^−2^, and found that 10 wt % PTFE was the optimal content to give the best performance of MPL and PEMFC. Qi et al. [75] also studied the influence of the cathode composition on the performance of PEMFCs, leading to the conclusion that the optimal cathode composition was 35% PTFE—65% conductive carbon black, while the composition of 45% PTFE—55% conductive carbon black showed the worst performance. Chen et al. [76] applied a method for measuring thermal conductivity of GDL materials based on fiber grating (FBG) sensing technology to study the influence of different PTFE contents on the performance of PEMFCs. They reported that when the content of PTFE was high, the thermal conductivity of the GDL decreased with the increasing pressure. However, when the PTFE content was low, the thermal conductivity increased with the increasing pressure. Moreover, the thermal conductivity of the GDL with an appropriate PTFE content was higher than that without PTFE. This method provides a new approach for monitoring the change of material properties in PEMFCs.

**Figure 6:**
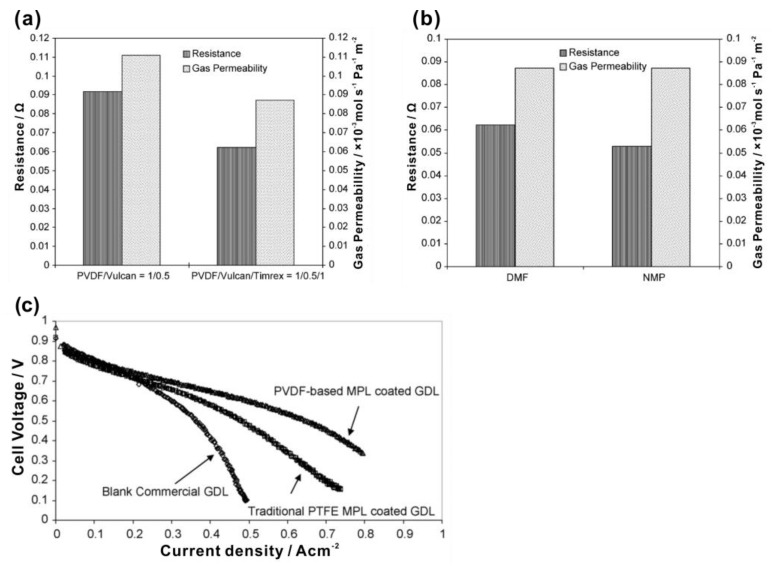
The resistance and gas permeability of (**a**) the MPL with PVDF-carbon black (Vulcan) added with and without electrically conductive filler (Timrex), and (**b**) the MPL using the PVDF solvent of DMF and NMP. (**c**) I–V curves of blank commercial GDL, traditional PTFE MPL-coated GDL and PVDF-based MPL-coated GDL. Reproduced with permission [72]. Copyright 2008, Elsevier.

Aside from PTFE, some other hydrophobic agents were applied. Gola et al. [71] applied PFPE peroxide (0.24–1.03 wt%) as the hydrophobic agent, and reported that the thermal decomposition of PFPE bonded covalently with the unsaturated part on the surface, making the MPL obtain super-hydrophobicity and an improved thermal stability, chemical stability and conductivity. They suggested that the performance of a PFPE-functionalized MPL is better than that using PTFE, enabling the MPL to cover a wide temperature and relative humidity range. Ong et al. [72] prepared a MPL added with PVDF and analyzed its structural and physical properties such as resistance, permeability and microstructure. The results indicated that the properties of the MPL were affected by the preparation parameters, such as PVDF concentration, the type and content of the conductive filler, the ratio between PVDF and conductive filler and the type of solvent for PVDF. As shown in Figure 6a, by adding conductive filler graphite (Timrex), the resistance of the MPL decreased, but the permeability also unfavorably decreased due to the slightly thickening of the MPL by adding Timrex. Figure 6b shows that the permeability of the MPL changed little by using different PVDF solvent, but lower resistance was obtained by using N-methyl-2-pyrrolidone (NMP) than N, N-dimethylformamide (DMF), since the MPL casted from the DMF slurry showed a more compact cross-section structure. Furthermore, the mass transfer was improved by using PVDF instead of PTFE, as shown in Figure 6c. Latorrata et al. [77] used FEP as the hydrophobic agent to impart the MPL with superior hydrophobicity, leading to reduced mass transfer resistance.

**Figure 7:**
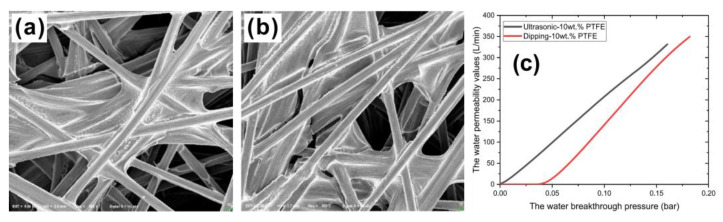
SEM images of the samples prepared with (**a**) the ultrasonic dispersion method, and (**b**) the direct dipping method. (**c**) Performance curves plotted with the water permeability values against water breakthrough pressure. Reproduced with permission [78]. Copyright 2022, Elsevier.

**Figure 8:**
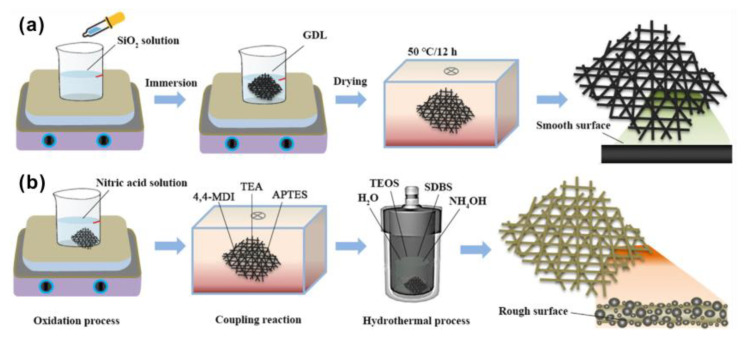
Schematic of the hydrophobic treatments of the GDL samples with (**a**) immersion and (**b**) hydrothermal methods. Reproduced with permission [80]. Copyright 2021, Elsevier.

In addition to the hydrophobic effect, effort was also dedicated to improving the drainage performance. Liu et al. [78] prepared a GDL with an ultrasonic dispersion technique to increase the contact angle to 141.97°, which is higher than that prepared by a direct dipping method. Furthermore, the ultrasonic dispersion technique also reduced the PTFE agglomeration, making the GDL more porous, as shown in Figure 7a,b. Along with this, as shown in Figure 7c, the water permeability was also improved. Li et al. [79] prepared a GDL which was added with CaCO_3_ and had a thickness of 240 μm. Two MPLs were deposited onto this GDL, helping enhance the hydrophobicity. Wang et al. [80] increased the surface roughness of the GDL via methods of immersion and hydrothermal treatment, as shown in Figure 8, leading to the increasing contact angle from 128 ± 1° (the GDL without treatments) to 146 ± 1° (immersion method) and 153 ± 0.4° (hydrothermal method).

Asides from the experimental works directly assessing the material properties, mathematical modeling and numerical simulation also suggest potential hints guiding the further development of the GDL. Here, some representative results are summarized in Table 1, showing the progress on improving the drainage performance of the GDL by theoretical calculation in recent years.

## 6. Structural Parameters for GDL

### 6.1. Pore Structure

The water management is one of the main functions of the MPL, whose porosity and pore size distribution impose significant influence. Chun et al. [47] regulated the condition for drying to change the distribution of pore size in the MPL, and found that higher drying temperatures led to rapid decomposition of the pore-forming agent, resulting in the formation of macropores. By lowering the drying temperature, the decomposition of pore-forming agent slowed down, giving rise to the formation of more micropores. The micropores showed a better capillary effect to remove water, while the macropores can provide better gas transport. So, a proper combination and distribution of the pores with different scales is necessary to optimize the performance of the MPL. Furthermore, different humidity conditions during the operation of PEMFCs also ask for different pore distribution. Hou et al. [93] established a three-dimensional Boltzmann model to simulate the porous transport layer by adjusting the porosity and distribution of pore size in MPL. It was found that even with the same porosity, the distribution of pore size influences the water transport significantly, suggesting that the porosity and distribution of pore size must be carefully determined to match the working conditions.

**Figure 9:**
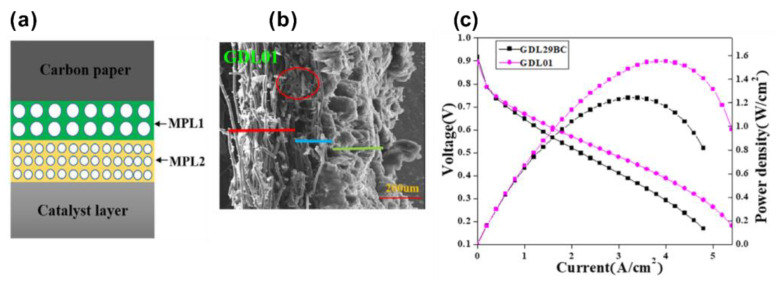
(**a**) A schematic diagram of the structure of the MPL with a gradient pore size (GDL01). (**b**) SEM images of MPLs without (GDL29BC) and with gradient pore size (GDL01). (**c**) I–V curves and power density of PEMFCs implemented with MPLs of GDL29BC and GDL01 performed under a relative humidity of 60%. Reproduced with permission [94]. Copyright 2020, John Wiley and Sons.

Lin et al. [94] prepared a gradient double-layer MPL structures by using conductive carbon black with different particle size, so as to achieve gradual change of pore size from GDL to CL, with the aim to improve the water management ability. They therefore designed a GDL with an MPL containing two layers that were different in pore size (as shown in Figure 9a) by using two types of conductive carbon blacks—one is acetylene carbon black (7–20 μm), the other is Vulcan XC-72 (20–100 μm). As shown in Figure 9b, the therefore prepared MPL (named as GDL01 in their work) has two layers (highlighted by blue and green lines), and the PEMFC with a gradient distribution of pore size showed a better performance than that with a uniform distribution of pore size, as shown in Figure 9c, attributed to the enhanced ability of water management by constructing a gradient MPL.

### 6.2. Thickness

**Figure 10:**
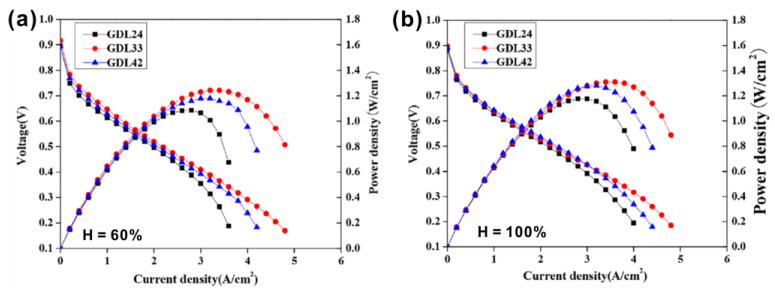
I–V curves and power densities of PEMFCs using GDLs containing bilayer MPLs with a controlled thickness for each MPL. The test was performed under the relative humidity of (**a**) 60% and (**b**) 100%. Reproduced with permission [96]. Copyright 2020, John Wiley and Sons.

MPL must has proper thickness to regulate the water balance between the CL and GDL. If the MPL is too thin, it cannot collect water efficiently from the CL. Tseng et al. [95] compared the performance of three PEMFCs with different MPL thicknesses and found that the MPL with a thickness of 84 μm showed the best performance over a certain range of current density (300–1300 mAcm^−2^). They also studied the influence of the distribution of pore size in MPLs with three different thicknesses and found that when the MPL was too thin and the pore size was too small, the gas transport was hindered. On the other hand, when MPL was too thick, the diffusion path turned to be too long, resulting in greater resistance. Therefore, it can be concluded that the MPL with an appropriate thickness is essential to ensure the overall transport of gas and water. Lin et al. [96] investigated the effect of the MPL thickness on the performance of PEMFCs. They prepared a bilayer MPL by spraying two slurries containing the conductive carbon black with a different size. It was found that the performance of the single cell changed with the adjustment of the thickness of the two MPL layers (MPL1 and MPL2). The authors tested the performances of three fuel cells using GDL24 (thickness of MPL1: 20 ± 2.0 μm, MPL2: 40 ± 1.6 μm), GDL33 (MPL1: 30 ± 1.5 μm, MPL2: 30 ± 2.5 μm) and GDL42 (MPL1: 40 ± 1.8 μm, MPL2: 20 ± 3.1 μm). As shown in the Figure 10, the fuel cell using GDL33 had the best performance at high current density under two different relative humidity conditions. This is because the distribution of the pore size was the optimal in GDL33 by adjusting the thickness of the two MPLs.

## 7. Other Attempts to Improve Performance of GDL

To further elevate the performance of PEMFCs, the properties of the GDL need to be studied and improved. Mathur et al. [97,98] investigated the influence of parameters for processing (e.g., molding and carbonization) on the properties of carbon fiber paper. Wang et al. [99] prepared a novel GDL with arrayed grooves, increasing the maximum power density by about 5.6%. The current density in the plate near the exit was increased as well, which was conducive to the stable operation of fuel cells. Chen et al. [100] modified a two-fluid model and studied distribution of the saturation of liquid water. Compared with the traditional two-fluid model, the liquid water saturation in PEMFCs with the baffle flow channel in their model was decreased significantly by taking the Forchheimer’s inertial force shock into account. Moradizadeh et al. [101] prepared a nickel mesh-based double layer GDL containing reduced graphene oxide (rGO) by printing the slurry onto the Ni mesh and found that the addition of rGO improved almost all the transport properties of GDL, but the electrical conductivity was not increased.

**Figure 11:**
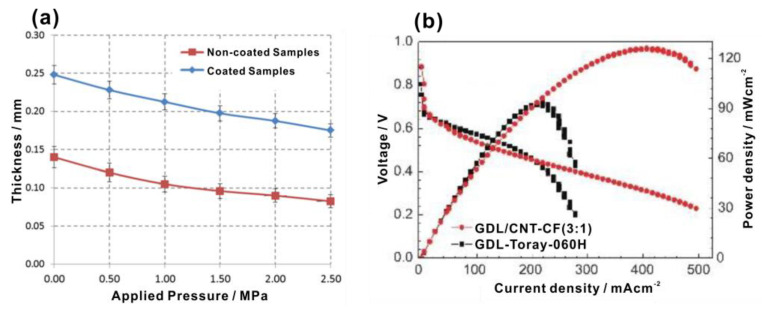
(**a**) Correlation between the thickness of the GDL and applied compressional pressure within 0.5 and 2.5 MPa. The data points on the top represent the GDL containing MPL and the data points at the bottom represent the GDL without MPL. Reproduced with permission [103]. Copyright 2013, Elsevier. (**b**) I–V curves and power density of fuel cells using GDL/CNT-CF (3:1) and GDL-Toray-060H as GDL. Reproduced with permission [104]. Copyright 2021, Elsevier.

One of the purposes to insert MPL between MPS and CL is to improve the contact between GDL and CL to reduce the contact resistance. Besides, clamping pressure also affects the contact resistance and therefore the performance of PEMFCs [102]. Bates et al. [103] studied the correlation between the thickness of the GDL and the applied pressure, and found that with the pressure increasing from 0 to 2.5 MPa, the thickness of the GDL decreased at an almost constant rate but the speed slowed down slightly after increasing the pressure over 1 MPa, as shown in Figure 11a, implying a potential strategy to precisely regulate the thickness and the contact resistance by applying proper pressure. Shu et al. [104] applied carbon fibers as the backbone and combining with highly-dispersed multi-wall CNTs by vacuum filtration after high-speed dispersing to prepare a GDL (known as GDL/CNT-CF). As shown in Figure 11b, the fuel cell using such a GDL showed superior performance over that using a conventional GDL (GDL-Toray-060H).

**Figure 12:**
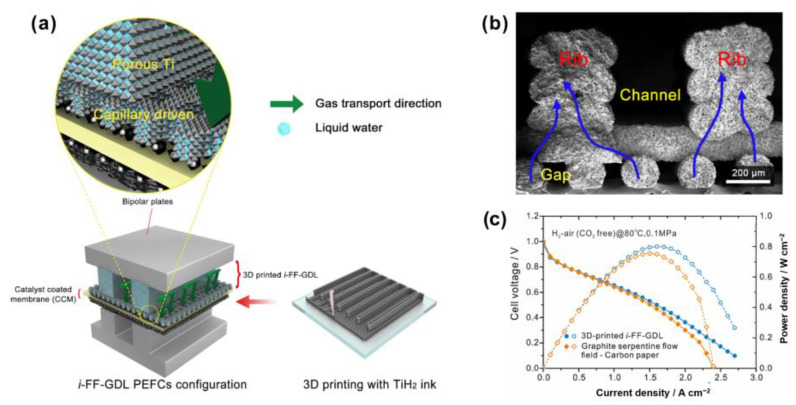
(**a**) Schematic of the 3D-printed GDL, (**b**) a SEM image of the microstructure of the i-FF-GDL, and (**c**) comparison of the performance of the fuel cells using 3D-printed i-FF-GDL and traditional anode configuration. Reproduced with permission [105]. Copyright 2021, Elsevier.

**Figure 13:**
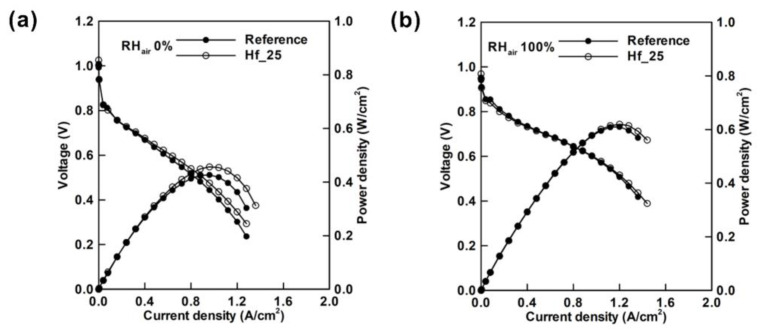
I–V curves and power densities of the PEMFCs with a reference GDL and that deposited with HfO_2_ for 25 cycles (Hf_25) fed with hydrogen (RH 50%) and air with a relative humidity of (**a**) 0% and (**b**) 100%. Reproduced with permission [106]. Copyright 2021, Elsevier.

Wang et al. [105] improved the mass transport by using 3D printing technique (Figure 12a). They printed an integrated flow field-gas diffusion layer (i-FF-GDL) with 85.3 wt% TiH_2_ and polymer ink. The microstructure is shown in Figure 12b. Such an approach increased the peak power density by 15%, as shown in Figure 12c. Lim et al. [106] modified the MPL with HfO_2_ by atomic layer deposition (ALD), leading to the increase of peak power density by 7%, as shown in Figure 13a. Moreover, the peak power also increased—although slightly—by 1.6% by feeding the air with 100% RH (Figure 13b), indicating that the modified GDL also improved the performance of the GDL in high RH conditions. Some other representative progress on improving the performance of the GDL is listed in Table 2.

## 8. Concluding Remarks

GDL is a key component in PEMFCs, providing physical and electrical connection between the CL and BP. The matrix of GDL is MPS, which in most cases uses carbon fiber paper or cloth to serve as mechanical support to MEA and construct routes for electrical conduction and gas/water transport between the CL and BP. However, since the pore size of the carbon paper and cloth is relatively large (tens of micrometers), and the electrical conductivity of the catalyst layer is low, it is difficult to form compact interface between the CL and MPS, resulting in high contact resistance. To solve this problem, an MPL with a small pore size—preventing penetration and thereby loss of catalyst particles—is added between the MPS and CL to improve the interfacial contact. Another function of MPL is draining water forming at the cathode CL, asking for proper hydrophobization process on MPL.

To further improve the performance of the GDL, the properties of both MPS and MPL must be optimized. Besides regulating the pore size and thickness, an interesting strategy is to construct a porous structure in the MPL—maybe also in the MPS—with a gradient change in pore size along the direction of thickness. Although there are some studies revealing the correlation between the pore size and the transport properties of the MPL, how the gradient pore size effecting the transport properties of the MPL is still lacking investigation. Furthermore, the optimal combination of pores (including mixing and gradient distribution) also needs systematic investigation. Some works interestingly reported that by using different conductive carbon materials, the MPL shows different hydrophobicity, which was simply attributed to the difference in the effective area of the pores [54]. However, more detailed analysis on the surficial properties of these conductive carbon materials and more insight into the difference in hydrophobicity is expected and may offer potential design strategies to precisely control the hydrophobicity of the MPL. Moreover, the conductive carbon materials are added into the MPL to improve the electrical conductivity. It is worth noting that in addition to the common additives such as carbon black, some other novel additives with high electrical conductivity (e.g., graphene) deserves an attempt, with their influence on the electrical and transport properties of the MPL being investigated in detail. For the MPS, due to the special structure of the carbon paper and cloth, it shows higher in-plane conductivity than it does through-plane conductivity. Modifying the process to fabricate the carbon paper and cloth, including optimizing or developing proper binders, may lead to the improvement of the through-plane conduction. Furthermore, due to the lamellar structure of MEA, there are several interfaces (e.g., MPS/MPL and MPL/CL). Although the contact resistance (which has been lowered) benefitted from the continuous development in this area, further effort on improving the interface is still necessary to boost the performance of PEMFCs.

## Figures and Tables

**Table 1:** Summary of recent theoretical results using the methods of computational fluid dynamics (CFD), volume of fluid (VOF), finite volume (FVM), lattice Boltzmann (LBM) and pore-scale model (PSM).

Authors	Method	Focus	Conclusion
Fan et al. [81]	CFD, VOF	Pore shapes and pore distance.	The pentagon and the hexagon pore structure decrease the droplet volume, pressure drop and cycle time. When pore distance is >0.6 mm, pressure drop increases sharply.
Kanchan et al. [82]	3D single-phase isothermal model	Stepwise, sinusoidal, and logarithmic non-uniform porosity configurations.	When logarithmic porosity decreasing configuration occurs in GDL, power density, current density and average diffusion coefficient reach the maximum.
Anyanwu et al. [83]	FVM, VOF	Compression ratio and fiber diameter	The effect of 10% compression ratio (CR) on liquid water saturation drop is relatively small (~8%). The effect of fiber diameter difference on water transport under compression is limited.
Wang et al. [84]	LBM	Linear type, stepped type and Transitional type non-uniform porosity configurations.	Linear porosity gradient distribution gives higher permeability than the others, and the maximum permeability is increased by 26.33%.
Wang et al. [85,86]	LBM	Binder and compression ratio.	The increase of volume fractions of binders (BVF) and CR reduce the permeability but increase the electric conductivity.
Liao et al. [87,88]	LBM	Diameter of the carbon fiber, porosity and thickness.	Diffusion characteristics of the GDL does not change obviously with the diameter of the carbon fiber and thickness increasing. However, the porosity increasing from 60 to 80% benefits the diffusion characteristics and leads to an increasing water saturation in GDL by 198.92%.
Zhu et al. [89]	PSM, LBM	Compression ratio.	Using 20% CR gets the best performance, considering gas diffusivity, effective electric and thermal conductivities.
Xie et al. [90]	VOF	Flow channel.	The flow channel with 50 μm in depth, 50 μm in radius and 200 μm in spacing has good drainage performance.
Liu et al. [91]	VOF	Flow channel.	The hydrophilic pipe with 400 μm in height, 37.5 μm in radius and 300 μm in spacing has good drainage performance.
Ira et al. [92]	LBM	Hydrophilic fibers percentage and compression ratio.	Using 10% hydrophilic fibers and 10% CR decreases the saturation level by 5.2% and shortens the time to reach steady-state by 22%.

**Table 2:** Summarization of improvement of GDL in recent years.

Authors	Approach	Contact Angle	Pore Size Distribution	Electrochemical Performance	Advantages
Lim et al. [106]	Using ALD to modify GDL by depositing HfO_2_ onto MPL	Reference GDL: 155°; GDL deposited with HfO_2_ (HF_25): 137°	Distribution into two regions: 10–25 μm and 0.05 μm	The peak power density is improved by 7% in low RH atmosphere and 1.6% in high RH atmosphere.	Performance of PEMFC in low humidity is improved.
Liu et al. [107]	adding PAN into the MPL	No PAN: 144°; 1 wt% PAN: 136.46°; 3 wt% PAN: 125.59°	Distribution into two regions: 20–70 nm and 0.06–0.1 nm	The peak power density of PEMFCs without PAN and containing 3 wt% PAN are 0.480 and 0.616 Wcm^−2^, respectively in low RH.	The performance of PEMFC in low PH is improved by adding PAN into the GDL.
Wang et al. [105]	GDL 3D-printed with TiH_2_ added.		-	In high current density region (>1 Acm^−2^), the peak power density increases by 15% and 8% by using i-FF-GDL under H_2_–O_2_ and H_2_-air (CO_2_ free) condition.	This 3D GDL separates gas and liquid flow channels by its “bone” structure
Fu et al. [108]	Carbon paper prepared by mixing FWCNT with short CF		-	The peak power density using new carbon papers is up to 365 mWcm^−2^, higher than 205 mWcm^−2^ for commercial carbon paper.	The heat treatment temperature (350 °C) is much lower than the traditional temperature (2000 °C). The flatness was improved to be <6.4 μm.
Wang et al. [109]	Dry-pressing method using CF and PVDF	139.4°	100–1000 nm	785 mWcm^−2^ in 40% RH, 1091 mWcm^−2^ in 100% RH.	The performance of PEMFC in low humidity is improved.
Navarro et al. [110]	Adding natural cotton to GDL	GDL with 40% cotton content: 170°.	GDL with 40% cotton content: 11,000–13,000 nm	The in-plane electrical conductivity of the GDL with 40% cotton is close to 4421 ± 160 Sm^−1^.	Low cost

## Data Availability

Not applicable.

## Abbreviations

AFCs	Alkaline fuel cells	MEA	Membrane electrode assembly
ALD	Atomic layer deposition	MPL	Microporous layer
BPs	Bipolar plates	MPS	Microporous substrate
BVF	Volume fractions of binders	NMP	N-methyl-2-pyrrolidone
CF	Carbon fiber	ORR	Oxygen reduction reaction
CL	Anode catalyst layer	PAFCs	Phosphoric acid fuel cells
CNF	Carbon nanofiber	PAN	Polyacrylonitrile
CNTs	carbon nanotubes	PDMS	Polydimethylsiloxane
CR	Compression ratios	PECVD	Plasma enhanced chemical vapor deposition
CVD	Chemical vapor deposition	PEMFCs	Proton exchange membrane fuel cells
DMF	N, N-dimethylformamide	PFPE	Perfluoropolyether
FCVs	Fuel cell vehicles	PI	Polyimide
FEP	Fluorinated ethylene	PTFE	Poly tetra fluoroethylene
FWCNT	Few-walled carbon nanotube	PVDF	Polyvinylidene fluoride
GDL	Gas diffusion layer	PVP	Polyvinyl pyrrolidone
HOR	Hydrogen oxidation reaction	rGO	Reduced graphene oxide
i-FF-GDL	Integrated flow field-gas diffusion layer	RH	Relative humidity
MCFCs	Molten carbonate fuel cells	SOFCs	Solid oxide fuel cells
MEA	Membrane electrode assemblies		
